# Adenocarcinoma of the colon and rectum in the Kashmiri population

**DOI:** 10.4103/0971-6866.60192

**Published:** 2009

**Authors:** A. Syed Sameer, Nissar A. Chowdhri, Mushtaq A. Siddiqi

**Affiliations:** Department of Clinical Biochemistry, Sher-i-Kashmir Institute of Medical Sciences, Soura, Srinagar, Kashmir - 190 011, India; 1 Department of General Surgery, Sher-i-Kashmir Institute of Medical Sciences, Soura, Srinagar, Kashmir - 190 011, India; 2 Department of Immunology and Molecular Medicine, Sher-i-Kashmir Institute of Medical Sciences, Soura, Srinagar, Kashmir - 190 011, India

Kashmir valley located in the northern division of India, surrounded by Himalayas has an unique ethnic population living in a temperate environmental conditions having distinctive food habits which play an overwhelming role in the development of GIT cancers over the genetic factors.[1-3] The food habits include consumption of sun-dried and smoked fish and meat, dried and pickled vegetables, red chilly, Hakh (a leafy vegetable of Brassica family), hot noon chai (salted tea) and Hukka (water pipe) smoke.[4] As previously reported,[5] the etiology and incidence of various GIT cancers in this population has been attributed to a probable exposure to nitroso compounds, amines and nitrates reported to be present in these local food stuffs.

Colorectal cancer being the commonest cancer, is the major cause of mortality and morbidity worldwide, there are nearly one million new cases of colorectal cancer diagnosed world-wide each year and half a million deaths.[6] The incidence of this malignancy shows considerable variation among racially or ethnically defined populations in multiracial/ethnic countries. Kashmir has been reported by now as a high-incidence area of GIT cancers.[1,3,4] Colorectal Cancer in Kashmir valley is the third most common GIT cancer after esophageal and gastric as per SKIMS Medical record registry, with almost 124 cases reported in 2009.

Here we have presented three cases of colorectal cancer in form of images, obtained from the patients who visited Department of General Surgery of SKIMS, for the resective surgery for the immediate treatment of the cancer.

**Figure 1:**
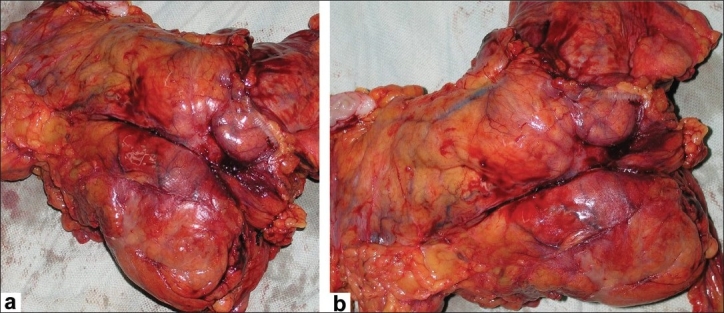
(a and b) Images showing the large tumor arising from the splenic flexure of the colon and infiltrating beyond the serosa into the pancreas and omentum. The tumor was classified as T4N2Mo. Histopathology revealed the tumor to be a well-differentiated adenocarcinoma

**Figure 2:**
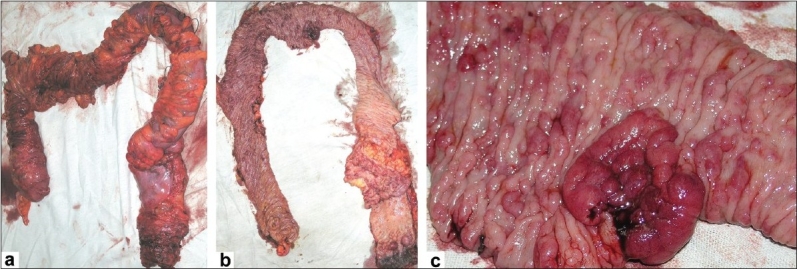
(a) Image showing the entire colon of the third-generation Familial Adenomatous Polyposis (FAP) patient, which was removed by Abdomino Perineal Resection (APR) Method. (b) Image showing the inner lining of the colon, revealing hundreds of polyps all over the surface of the colon and a rosette-shaped malignant tumor at the transverse section of the colon. (c) A close-up of the rosette-shaped malignancy of the colon. The tumor was classified as T4N2M1. Histopathology revealed the tumor to be poorly-differentiated adenocarcinoma

**Figure 3:**
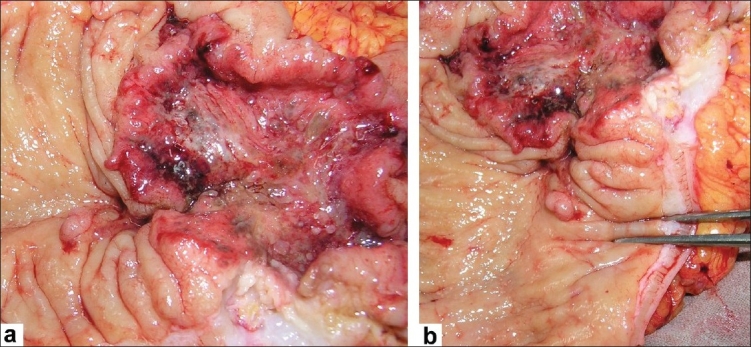
(a and b) Images showing the tumor of the colon, located about 10 cm from the anal verge and the sessile single polyp. The tumor was classified as T4NoMo. Histopathology revealed the tumor to be well-differentiated adenocarcinoma
